# Distributed Fiber Optic Strain Sensing Technology for Monitoring Soil Deformation Induced by Leakage in Buried Water Pipelines: A Model Test Study

**DOI:** 10.3390/s25020320

**Published:** 2025-01-08

**Authors:** Lin Cheng, Yongkang Sun, Zhaohan Wang, Wenqi Gao, Zhuolin Li, Zengguang Xu, Jiang Hu

**Affiliations:** 1 State Key Laboratory of Eco-Hydraulics in Northwest Arid Region, Xi’an University of Technology, Xi’an 710048, China; 15030069531@163.com (Y.S.); m15191144652@163.com (W.G.); l2498470526@163.com (Z.L.); xuzengguang@xaut.edu.cn (Z.X.); 2 Xi’an Water Investment Company Limited, Xi’an 710000, China; wzh740320880@hotmail.com; 3 State Key Laboratory of Hydrology–Water Resources and Hydraulic Engineering, Nanjing Hydraulic Research Institute, Nanjing 210029, China; huj@nhri.cn

**Keywords:** leakage of underground water pipeline, distributed fiber optic strain sensing (DFOSS), ground subsidence, model test

## Abstract

Water pipelines in water diversion projects can leak, leading to soil deformation and ground subsidence, necessitating research into soil deformation monitoring technology. This study conducted model tests to monitor soil deformation around leaking buried water pipelines using distributed fiber optic strain sensing (DFOSS) technology based on optical frequency domain reflectometry (OFDR). By arranging strain measurement fibers in a pipe–soil model, we investigated how leak location, leak size, pipe burial depth, and water flow velocity affect soil strain field monitoring results. The results showed that pipeline leakage creates a “saddle-shaped” spatial distribution of soil strain above the pipeline, effectively indicating ground subsidence locations. When only one survey line is arranged, it is preferable to place the optical fiber directly above the pipeline. Surface monitoring fibers primarily detected tensile strain, with more pronounced peak values observed under conditions of larger leak size, higher flow velocity, shallow burial depth, and top-pipe leakage location. Monitoring fibers below the pipeline showed mainly unimodal distribution, with peak strain coinciding with the leak location. The sequential timing of strain changes at different fiber positions enabled the determination of soil seepage direction. This study demonstrates that DFOSS technology can provide important support for the early warning of such geological disasters.

## 1. Introduction

To meet the domestic water and industrial water needs of people in urban areas, many water diversion and water transfer projects have recently been constructed, and many water transmission pipelines have been buried in many areas. After they are subjected to long-term use, the tensile and impact strengths are reduced because of the aging of the pipes, which can cause problems such as leakage and rupture. When buried water pipelines leak, water seeps through the soil pores, causing soil particles to lose their support and causing ground deformation. An analysis of numerous ground subsidence accidents revealed that more than half of the accidents were caused by the leakage of underground pipelines [1]. The deformation or subsidence of the soil around a buried water pipeline caused by the leakage of the buried water pipeline not only severely affects the normal use of the pipeline but also may endanger the life and property of local residents [2]. Therefore, it is essential to study monitoring technologies for soil deformation around buried water pipelines.

The study of the development patterns of soil deformation and ground collapse caused by the leakage of buried water pipelines is the basis for soil deformation monitoring. Some researchers have used the physical model test method. Guo et al. [3] investigated the problem of leakage from sewage pipes through an indoor model test and reported that the height of the water head and burial depth of the pipe strongly affected the shape and volume of the erosion zone. Sato et al. [4] analyzed the effects of underground structures on the seepage and erosion of sewage pipelines through indoor model tests and reported that underground cavities and local seepage developed along the shortest path from the surface to the damaged opening in the pipeline. Zheng et al. [5] explored seepage erosion in fine sandy soil strata and medium sandy soil strata through model tests and reported that as soil loss increased, the surface settlement profile exhibited an “inverted triangular” shape. Wang et al. [6] conducted indoor model tests to evaluate pipeline leakage under different loads and pipeline burial depths and reported that the deeper the pipeline is, the slower the soil settlement. Liu et al. [7] designed a set of visualized test setups and analyzed the erosion change characteristics of formation collapse induced by pipeline leakage under full flow conditions. Liu et al. [8] validated the proposed seepage diffusion model through experimentation; that is, with increasing saturated permeability coefficient, initial saturation, and internal pressure, the fluid diffusion distance increased. Liu et al. [9] constructed a seepage diffusion model for pipeline leakage fluid; analyzed the effects of the fluid self-weight, saturation permeability coefficient, initial saturation, and internal pressure on the diffusion distance of pipeline leakage fluid in unsaturated formations; and validated the model through experimentation. Dai et al. [10] designed a set of model test rigs to examine the evolution pattern of the soil erosion area and subsidence pits caused by the extravasation of groundwater pipelines and analyzed the formation process and influence of the cone-shaped erosion area above the pipeline defect. The erosion angle was not related to the initial water content of the sandy soil. The deeper the pipeline burial depth, the lower the water flow velocity and the smaller the erosion angle. Wang et al. [11] used standard sandy soil with uniform grading to analyze the effects of different leakage locations of pipelines on the development pattern of soil erosion. The researchers reported that pipeline leakage involves three stages: initial leakage, leakage development, and leakage convergence. Preliminarily, in the leakage stage, the surface had no significant settlement; in the seepage development stage, the surface slowly settled; and in the seepage convergence stage, stable soil arches formed at the seepage holes and the soil particles were no longer lost.

The commonly used soil deformation monitoring technologies include interferometric synthetic aperture radar (InSAR) [12,13], satellite positioning [14], wireless sensor networks (WSNs) [15], microseismic monitoring systems [16], laser technology [17], and digital image measurement technology [18,19]. Compared with the above-mentioned technologies, distributed fiber optic strain sensing (DFOSS) technology has the advantages of good real-time performance, distributed measurement, and minimal environmental influence, and can continuously monitor the deformation of the soil around a pipeline for a long period with high precision and good cost effectiveness. Distributed fiber optic monitoring technology has been applied in the fields of roof settlement [20], karst area subsidence [21], slope rock and soil monitoring [22], stratum monitoring [23], and permafrost deformation monitoring [24]. For the monitoring of soil deformation caused by the leakage of buried water pipelines, Wei et al. [25] conducted a ground collapse model test via the distributed fiber monitoring method. The results revealed that the fiber strain monitoring curve accurately reflected the deformation state of the overburdened soil at different stages of collapse. Cheng et al. [26] conducted an indoor model test to investigate the monitoring effect of distributed fiber strain sensing technology under different conditions in the monitoring of land subsidence caused by underground cavities. Li et al. [27] used fiber strain sensing technology to monitor the deformation of soil in a pipe–soil system. An analytical model that uses fiber strain measurements to calculate land subsidence is proposed. The above studies used water bladder pumping to simulate the cavity created by the leakage of the buried water pipeline and did not simulate the process of soil deformation caused by pipeline leakage.

This paper investigates DFOSS technology, which is based on distributed optical frequency domain reflectometry (OFDR). Model tests are conducted to analyze ground deformation caused by the leakage of buried water pipelines under various conditions, including different burial depths, flow velocities, leakage locations, and leak sizes. Section 2 describes the principle of OFDR strain sensing. Section 3 describes the design of the indoor model test of soil deformation caused by leakage of the buried water pipeline. Section 4 analyzes the test phenomena and test monitoring data. Section 5 discusses the types of ground collapse in the test and the coupling between the fiber and the soil.

## 2. Basic Principle of Soil Deformation Monitoring Based on the OFDR

**Figure 1:**
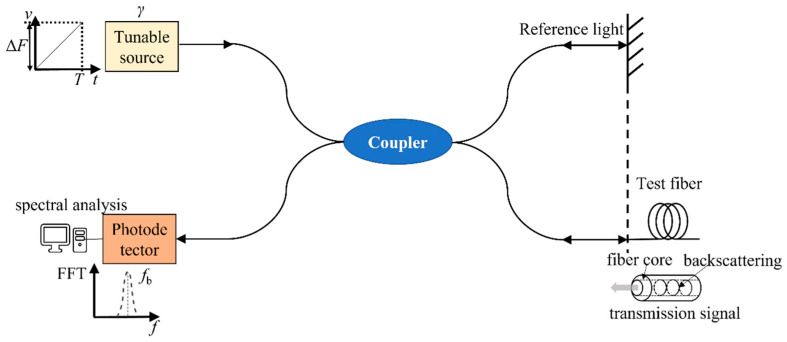
Principle of OFDR strain sensing.

Among the commonly used fiber distributed strain measurement technologies, spatial resolution technologies such as Brillouin optical time-domain reflectometry (BOTDR) and Brillouin optical time-domain analysis (BOTDA) are the most advanced. Because the spatial resolution is low, dm- or cm-level spatial resolution can only be achieved at short distances, and the long-distance resolution is reduced to the m level, which is suitable for long-distance strain or temperature monitoring with low accuracy requirements in actual water pipeline projects. Compared with the above-mentioned technologies, OFDR is a type of fiber sensing technology that is based on Rayleigh scattering, has extremely high spatial resolution and a high signal-to-noise ratio, and can be used for strain measurement in indoor model tests. The strain-sensing principle of OFDR is shown in Figure 1.

In Figure 1, *v* represents the frequency of the light source, Δ*F* represents the tuning range of the light source, *t* represents time, *T* represents the sampling time, *γ* represents the tuning rate of the light source, and *f* and *f*_b_ represent the beat frequency and maximum beat frequency, respectively, corresponding to the interference signal frequency. FFT is a fast Fourier transform. The coupler divides the continuous light emitted by the light source into two channels, with one beam used as reference light and another beam used as probe light, which is transmitted into the fiber being tested. When the probe light propagates forward in the fiber, Rayleigh scattered signals are continuously generated. This signal light and the reflected reference light pass through the coupler, undergo beat frequency interference, and are detected by the photodetector. When the strain at a certain point inside the fiber changes, the refractive index inside the fiber changes, and the corresponding frequency of the Rayleigh scattering signal also changes. When the frequency of the fiber backscattering signal is measured, the change in the external temperature field or strain field can be measured [28].

When the optical fiber is used to monitor the soil deformation caused by the leakage of the buried water pipeline, the beat signal light field of the main interferometer can be expressed as:

(1) $$I(t)=2E_{0}^{2}\sqrt{R_{z}}cos\;[2\pi \gamma \tau_{z}t+\phi_{0z}]$$

In the formula, *E*_0_ is the initial amplitude of the light source; *R_Z_* is the equivalent reflection coefficient of the Rayleigh backscattering of the fiber; *γ* is the scanning speed of the light source; $\tau_{z}$ is the time delay generated by the round-trip of light at a certain position z in the optical fiber; and $\phi_{0z}=v_{0}\tau_{z}-\gamma \tau_{z}^{2}/2$ is a time-independent term, which is related to the initial optical frequency *v*_0_ of the swept light source. Let $f_{b}=\gamma \tau_{z}$ denote the beat frequency of the fiber *z* position. When the linear sweep speed of the laser is a fixed value, there is a linear correspondence between the beat frequency value and the fiber position, so as to ensure the continuous distributed non-blind zone measurement of OFDR. The sensing spatial resolution can be expressed as:

(2) $$\Delta L=N•\Delta Z=N•\frac{\lambda_{c}^{2}}{2n\Delta \lambda }$$

where: Δ*Z* denotes the distance between adjacent positions after OFDR discretizes the fiber distance domain, which is inversely proportional to the wavelength scanning range Δ*λ* of the laser; *λ*_c_ is the central wavelength of the swept light source; and *N* is an integer number denoting the number of points of the additive window in the fiber distance domain.

The Rayleigh scattering spectral information at each position of the fiber to be tested is a steady-state signal. When the external environment changes, the backward Rayleigh scattering spectrum of the fiber will drift to a certain extent. The relationship between the strain Δ*ε* of the fiber and the wavelength drift *δλ* is as follows [29]:

(3) $$\Delta \varepsilon =\frac{\delta \lambda }{(1-P_{e})\times \lambda_{c}}$$

where *P*_e_ is the elastic optical coefficient. The relationship between material strain and stress is as follows [29]:

(4) Δσ=Δε×E

where Δ*σ* is the stress and *E* is the modulus of elasticity of the material.

**Figure 2:**
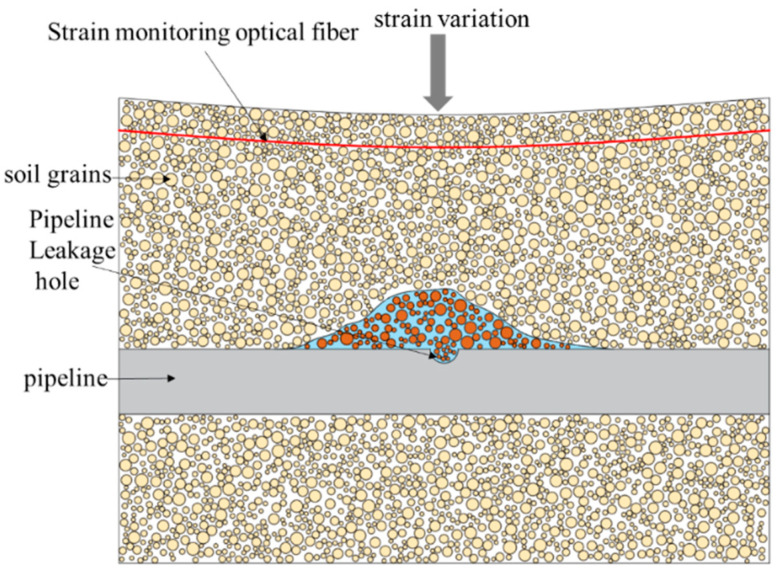
A schematic diagram of soil strain change caused by leakage of buried water pipeline.

When leakage from the buried water pipeline occurs, the soil near the seepage hole of the pipeline is saturated with water, and the water content of the soil changes, which changes the shear strength and internal friction angle and makes the soil more prone to deformation. When water flows through the soil near the pipeline seepage hole, the porosity of the soil changes, and the soil structure loosens, causing soil particle loss as shown in Figure 2. Since optical fibers are typically embedded directly within the soil and maintain close contact with it, the soil deformation is transmitted to the optical fiber through the contact interface, causing the fiber to undergo tensile or compressive strain. When the optical fiber experiences strain, it alters the phase and intensity of the Rayleigh backscattered light. By measuring the wavelength shift *δλ* in the Rayleigh scattering spectrum and utilizing the relationship between the strain Δ*ε* and the spectral shift *δλ*, the strain experienced by the optical fiber can be calculated. This enables real-time monitoring and precise localization of soil deformation.

## 3. Model Experimental Design

### 3.1. Test Equipment and Materials

The instruments and equipment used in this experiment include an OFDR demodulator, a water tank, a strain measurement fiber (optical fiber type: SMG.652b, coating type: polyimide elastic sheath, diameter: 0.9~1.5 mm, cable weight: 1.0 kg/km), a laser range finder, and a flow meter. The materials used in the experiments include loess, sandy soil, PVC pipes, and acrylic plates. Table 1 lists the main test equipment.

### 3.2. Test System Design

**Figure 3:**
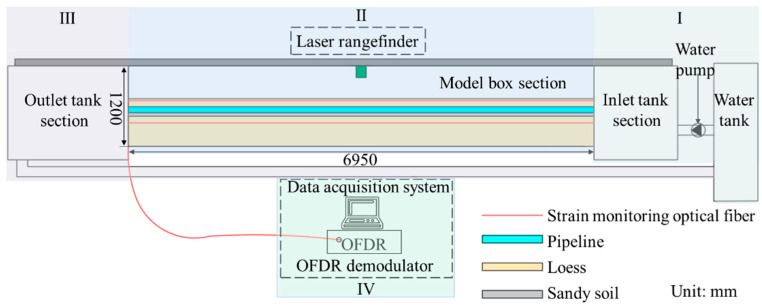
Schematic of the model test system for soil deformation caused by the leakage of buried water from a pipeline (front view).

The overall structure of the model test system, which uses DFOSS technology to monitor soil deformation caused by the leakage of the buried water pipeline, is shown in Figure 3. The system consists of four parts: the water inlet box (I), the model box (II), the water outlet box (III), and the data acquisition system (IV).

#### 3.2.1. Inlet Tank

**Figure 4:**
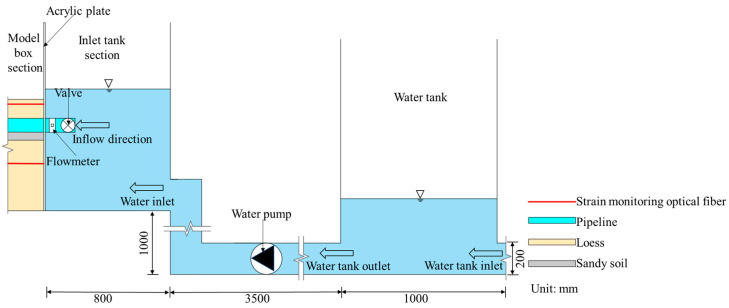
Detailed structure of the inlet tank and the model box (front view).

The inlet tank is used mainly to supply water to the water delivery pipeline and stabilize the water head in the inlet tank by changing the inflow rate of the water pump (before the test, the water head corresponding to each flow rate needs to be calibrated). The inlet tank section features a built-in acrylic plate with a thickness of 1 cm. The water inlet end of the water delivery pipeline passes through the round hole of the acrylic divider and is sealed with glass glue to prevent leakage. Moreover, this divider separates the water inlet tank section from the model box section. The detailed structure is shown in Figure 4.

#### 3.2.2. Model Box

The model box is the core part of the entire test device and is used to simulate the buried water pipeline and the soil around the pipe. The strain measurement fiber optic cable and the laser range finder are arranged in the soil around the pipe to monitor the entire deformation process caused by pipeline leakage.

**Figure 5:**
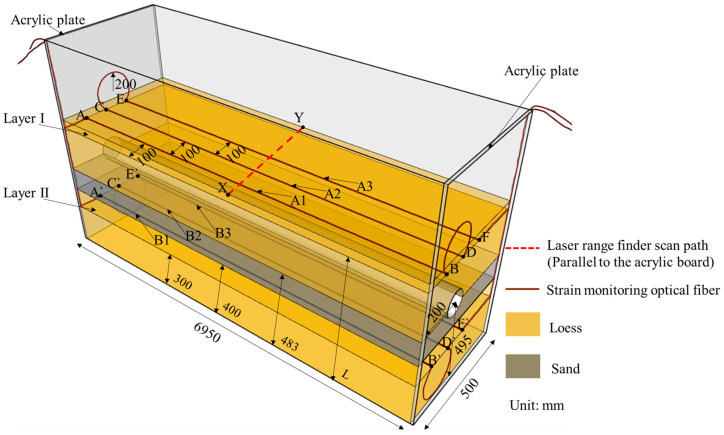
Layout of the test instruments. (A, B, C, D, E, F, A`, B`, C`, D`, E`, F` are the intersection points of optical fiber perpendicular to acrylic plate. X and Y are the intersection points of the scanning path of the laser range finder and the boundary of the model box.).

The model box was based on the buried water pipeline in a long-distance water diversion project. The length scale of the buried water pipeline and its surrounding soil was 1:34. The upper part of the model box was not sealed to facilitate the measurement of ground settlement. Figure 5 shows the test instrument setup.

For the laying of fibers, this paper refers to the previous research [25,26] and considers the arrangement of the optical fiber in the soil under the pipeline, so as to compare and analyze the monitoring effect of the optical fiber at different positions. The model box is divided into two layers along the horizontal direction, namely, layer I and layer II, which are used to monitor the soil deformation during the test. The water delivery pipeline was simulated by using a PVC pipe with an outer diameter of 9 cm. The distance between the axis of the pipe and the left sidewall of the model box was 20 cm, and the neutral plane of the pipe was 49.5 cm from the bottom plate of the model box.

(1) Arrangement of the strain measurement fiber:

① Surface deformation measurement. The fiber for surface deformation monitoring was pre-embedded. The fibers were embedded in the loess while the loess was being filled. A certain pretensioning strain was applied to the fiber. The strain value of the fiber in the initial pretension state was zero. In this test, the distance L between the horizontal optical fiber for surface deformation monitoring and the floor of the model box varied depending on the test conditions, but the burial depth of the optical fibers was always 3 cm, and the fibers were arranged in an “S” shape. The middle measuring section was located directly above the tube axis, and the other two sections were positioned on either side, each spaced 10 cm apart. The fiber turning radius was 20 cm, and the distance from the fiber to the glass wall was 10 cm.

② Deformation measurement of the soil under the pipeline. In the test, the horizontal fiber for soil deformation measurement in the lower part of the pipeline was set up 15 cm below the bottom of the pipeline, 10 cm from the interface between the loess foundation and the sandy soil cushion, 15 cm from the interface between the sandy soil cushion and the overburden of loess, and 30 cm from the bottom plate of the model box. The horizontal fiber was also pre-buried, and the arrangement was the same as that of the horizontal optical fiber for surface deformation measurement.

**Figure 6:**
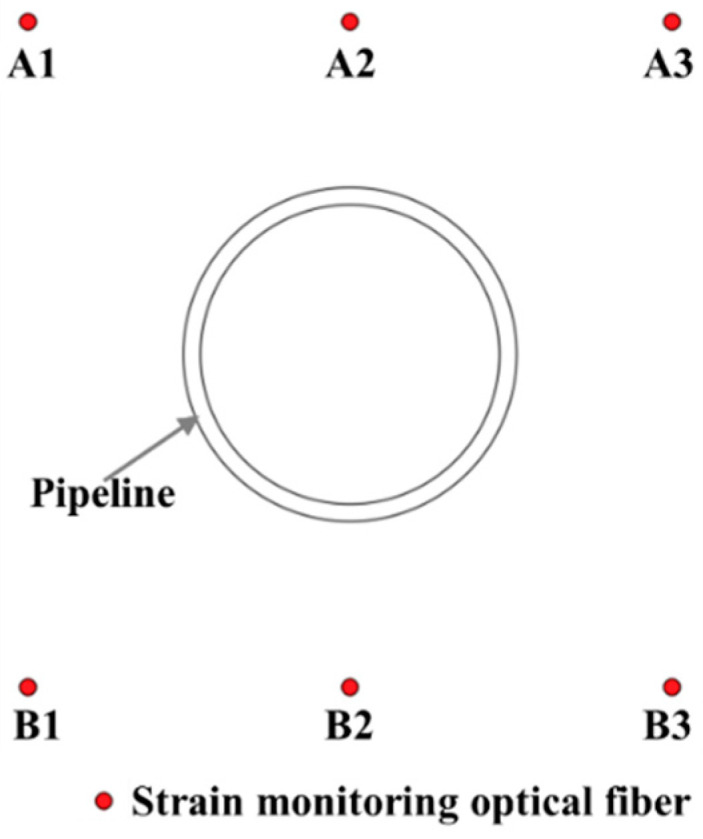
Cross-sectional view of the relative positions of the fiber measuring section and the duct (right view).

In the test, the lengths of both the surface deformation monitoring fiber and the soil deformation monitoring fiber at the lower part of the pipeline was 26.5 m, where 0 m is the connection between the fiber and the OFDR interrogator, 2.5 m is the position where the fiber enters the model box, and the length of the fiber exiting the model is 25.65 m. The monitoring fibers above the pipeline, which are the surface deformation monitoring fibers, are named A1, A2, and A3. The monitoring optical fibers under the pipeline are the monitoring optical fiber for the deformation of the soil under the pipeline, which are named B1, B2, and B3. The relative locations of the fiber measuring sections and the pipeline are shown in Figure 6.

(2) The laser rangefinder is fixed on the fabricated support, which is also installed on the slide rails, to measure the settlement of the soil surface under different conditions.

#### 3.2.3. Outlet Tank

**Figure 7:**
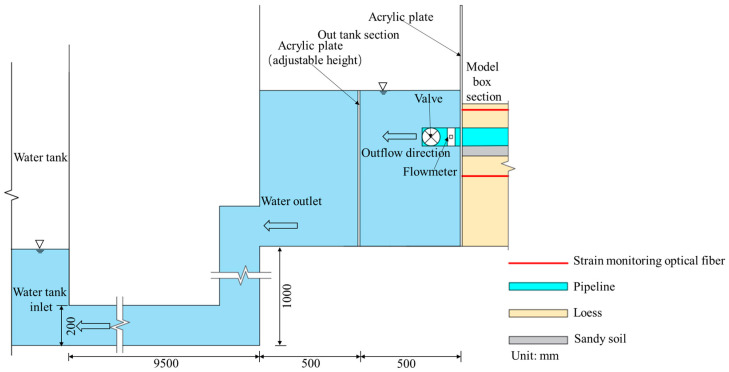
Detailed structure of the outlet tank and the model box (front view).

The outlet tank is used mainly to collect water and sediment discharged from water pipelines. Two acrylic plates, each 1 cm thick, are built into the outlet tank section. After a hole is punched in the middle of one acrylic plate, the water delivery end of the water pipeline is passed through and sealed with glass glue. This partition plate also separates the water tank section from the model box section. The other acrylic plate is located 50 cm behind the water delivery end of the water pipeline. Its height can be adjusted according to different working conditions so that it is at the same height as the water head of the inlet tank. This ensures that the inside of the pipe is always in a state of full flow and constant pressure. The detailed structure is shown in Figure 7.

#### 3.2.4. Data Acquisition System

The data acquisition system, which includes the OFDR demodulator and laser range finder, is used to record the measurement of each monitoring instrument during the test and displays the data process line or image data.

### 3.3. Test Protocol

#### 3.3.1. Control Methods for the Test Influencing Factors

This test did not consider the effects of groundwater, rainfall, or other external loads. In the test, only the water pipeline leakage location, leak size, internal flow velocity, and pipeline burial depth were simulated. In the experiment, by changing these different test variables, the simulation of different working conditions was realized.

**Figure 8:**
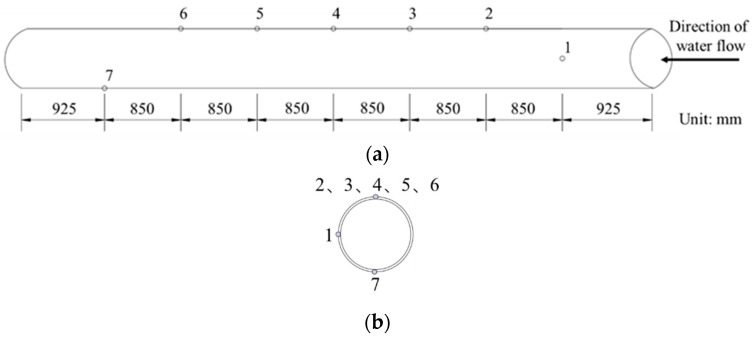
Pipe opening. (**a**) Front view of the duct opening location; (**b**) right view of the duct opening location.

(1) Leakage location and size: Drill bits of different sizes were used to drill holes at corresponding positions on the PVC water pipe to control the size of the leakage. The locations and sizes of the drill holes are shown in Figure 8. Pipeline damage in tests involves the use of round holes, which are located mostly at the top of the pipeline [7,30,31]. Therefore, in this test, holes 2–6 were located at the top of the pipe. Hole 1 was located at the waist of the pipe, 92.5 cm from the water inlet end of the pipe, with an opening size of 6 mm. Hole 2 was located 85 cm to the left of hole 1. The spacing between holes 2–6 was 85 cm, and the opening sizes were 2 mm, 4 mm, 6 mm, 8 mm, and 10 mm. Hole 7 was located at the bottom of the pipe, 92.5 cm from the water outlet end of the pipe, with an opening size of 6 mm. The tests were conducted in accordance with the order of the hole numbers. During the test, with the exception of the leakage hole that was opened under the corresponding working conditions, the remaining holes were sealed with rubber stoppers.

(2) Flow rate in the pipe: By changing the water level in the inlet tank, water heads of different sizes were provided to change the flow rate in the tube. In actual engineering, the flow rate of water in a pipe is generally 0.5–3.0 m/s [7], and pipe water pressure is generally more than 0.6 MPa. Taking into account the fact that the height of the test model cannot reach the height of the pipe water pressure corresponding to the height of the working head, and that the purpose of this paper is to study the different pressure in the pipe, the strain measurement fiber optic monitoring of soil deformation change rule, which is scaled according to the flow rate scale, was set to 0.171 m/s, 0.257 m/s, 0.343 m/s, 0.429 m/s, and 0.514 m/s, and the water head in the water inlet tank section was calibrated on the basis of the flow rate in the pipe.

(3) Pipeline burial depth: The burial depth of the pipeline was changed by changing the thickness of the soil above the pipeline. In actual projects, the burial depth of pipelines is 2–6 m [26]. In the experiment, the burial depths were set to 6 cm, 9 cm, 12 cm, 15 cm, and 18 cm.

In summary, the test conditions proposed in this experiment are shown in Table 2.

#### 3.3.2. Test Procedures

The specific steps of the test were as follows:

(1) Preparation and storage of test soil: Considering the special properties of loess, such as easy spalling, erosion, and its relatively loose nature, the potential of ground collapse caused by water pipeline leakage in a loess area is larger than that in an ordinary soil area, so loess was selected as the test soil. According to the requirements of the test, the sandy soil sample used in this experiment was composed of medium-coarse sandy soil (sandy soil passed through a 5 mm sieve) and loess at a mass ratio of 9:1. The loess soil sample was natural air-dried loess from a loess area. Because the loss of water from the soil sample would cause a decrease in the mass proportion of water and an increase in the mass proportion of soil particles in the fill mass required for each group of tests, resulting in differences in fill formation, the soil samples in this experiment were stored in sealed plastic bags to prevent water loss.

(2) Soil sample filling: First, the foundation loess was filled. According to the volume of the filled soil after compaction in each test, the quality of the loess that was needed to fill the model box under the corresponding density was calculated. The model box was filled with loess four times via the “falling feather method”. The foundation was 40 cm thick and filled in four layers. Each layer was 10 cm thick after being compacted by a spatula. Moreover, before the next layer of soil was backfilled, the surface of the soil needed to be roughened. The sand cushion was then filled, and the sand cushion at the bottom of the pipe was 5 cm thick. Then, the loess was continuously backfilled above the sand cushion so that the loess was flush with the top of the pipeline. When the upper loess of the pipeline was backfilled, multiple layers were filled. The first layer was 6 cm thick after being compacted by a spatula. Subsequently, each layer was compacted by a spatula and 3 cm thick. Before the next layer of soil was backfilled, the soil surface needed to be roughened. According to different test conditions, the samples were compacted to the corresponding thickness.

(3) Installation of monitoring instruments:

① The soil deformation measurement fiber in the lower part of the pipeline was laid after the soil sample was filled to a depth of 30 cm. Then, the fiber was laid on the loess surface, the fiber and the jumper cord were tightly fused, and the installation location of the fiber was accurately determined through the monitoring software (OSI) of the fiber instrument. After the installation was complete, a certain pretension was applied to the fiber to ensure that it had a certain prestress.

② The surface deformation measurement optical fiber was laid on the loess surface at the corresponding height, the fiber and jumper cord were then tightly fused, and the fiber installation location was accurately determined through the fiber instrument monitoring software. After the installation was complete, a certain pretension was applied to the fiber to ensure that it had a certain prestress.

③ The laser rangefinder was fixed on the support, and the support of the laser rangefinder was adjusted so that it could measure the collapsed area.

**Figure 9:**
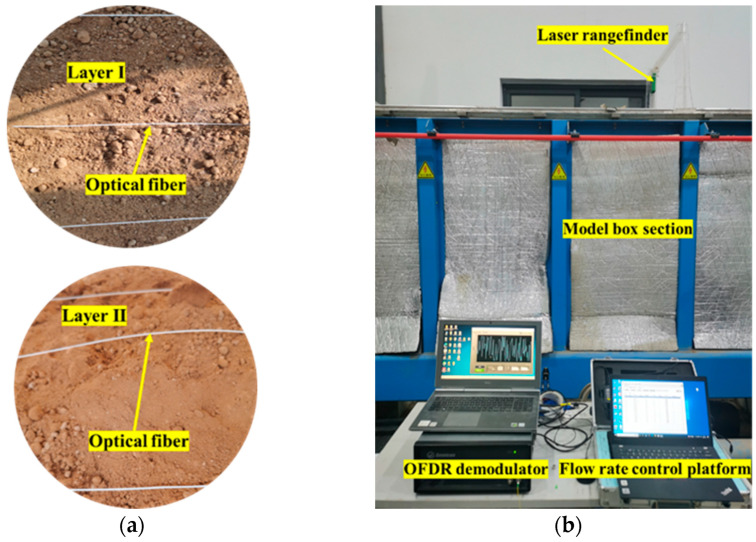
Test site layout. (**a**) Fiber arrangement; (**b**) instrument arrangement.

The layout of the test site is shown in Figure 9.

(4) Monitoring of ground subsidence caused by pipeline leakage: The test began after the installation of the monitoring instruments was completed. The water storage at the water outlet tank was started, and the water storage stopped when the water level reached the bottom of the pipe (the valve at the water outlet of the pipeline was always open). The water inlet tank section started storing water. When the water inlet tank section reached the set water level, the valve at the water inlet of the pipeline was opened to supply water to the pipeline. Simultaneously, the water supply time was used as the starting time to record the water supply duration and the flowmeters at both ends of the pipeline. The monitoring interval of the instrument was 1 min; that is, the fiber performs data acquisition every 1 min, and the laser rangefinder performs distance measurements every 1 min. The transparent glass wall was observed, and the test ended when the distance between the wetted range and the bottom plate of the model box was 10 cm. During the test, photos of the soil were continuously taken to monitor the changes in the soil structure during the test. After the end of the test, the water supply was stopped. After the water level in the water inlet tank section decreased, the water inlet valve was closed to drain the pipeline. In preparation for the next set of tests, steps (2)–(4) were repeated starting from hole no. 1; the tests were carried out in turn, and all working condition tests were completed.

(5) Data processing: According to the change in strain along the optical fiber obtained by the test, the strain curve in the test process was drawn and the influence of four factors—different burial depths of the pipeline, flow velocities in the pipeline, leakage positions, and leakage sizes—on the ground deformation law caused by the leakage of buried water from the pipeline were investigated. According to the data of the laser range finder obtained from the experiment, the land subsidence curves under different influencing factors were drawn.

## 4. Test Result Analysis

### 4.1. Experimental Phenomena

In this test, the deformation of the surface soil can be divided into two types: collapse with holes and collapse with no holes.

**Figure 10:**
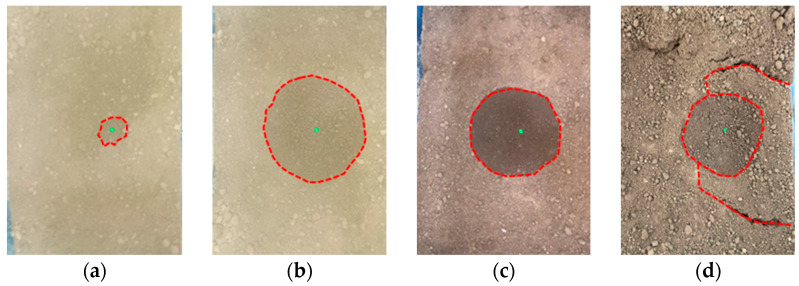
There was no holes in the surface soil (the green dots represent the locations of pipeline leakage, and the red dotted line represents the soil settlement area). (**a**) State at the 11th minute of Condition 2-5; (**b**) state at the end of Condition 2-5; (**c**) state at the end of Condition 3-1; (**d**) state at the end of Condition 4-1.

Collapse without holes occurred under working Conditions 2-5, 3-1, and 4-1. These are the cases where the pipelines are buried deeply, the flow velocity in the pipelines is low, and the leakage loss size is small, as shown in Figure 10. The deformation of the surface soil during the tests was similar. Under working Condition 2-5, the surface soil did not have any holes during the test. After approximately 11 min, the surface soil appeared collapsible, accompanied by the generation of fine axial cracks, as shown in Figure 10a. Owing to the deep burial of the pipeline, there was no piping phenomenon, and no water flow was observed in the surface soil. As the test progressed, a circular settlement pit centered on the leakage point appeared.

**Figure 11:**
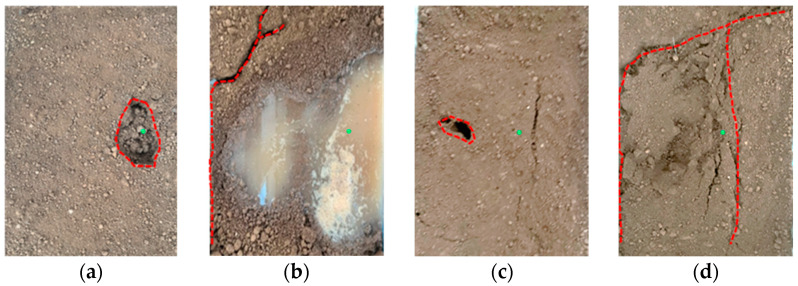
Holes suddenly appeared in the surface soil and collapsed (the green dots represent the locations of pipeline leakage, and the red dotted line represents the soil settlement area). (**a**) State at the appearance of the hole under Condition 1-1; (**b**) state at the end of Condition 1-1; (**c**) state at the appearance of the hole under Condition 1-3; (**d**) state at the end of Condition 1-3.

**Figure 12:**
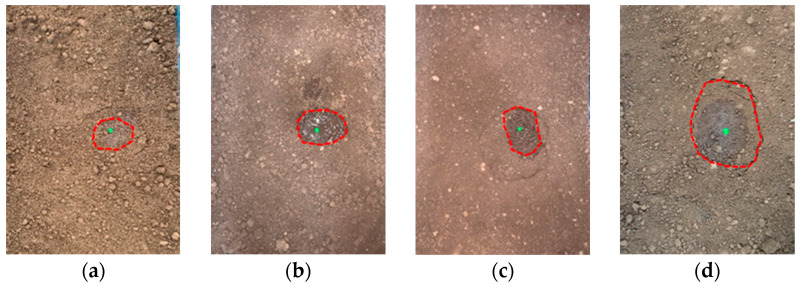
The surface soil wetted a small area before it formed holes and collapsed (the green dots represent the locations of pipeline leakage, and the red dotted line represents the soil settlement area). (**a**) State at the collapse under Condition 1-2; (**b**) state at the collapse under Condition 3-3; (**c**) state at the subsidence under Condition 4-2; (**d**) state at the subsidence under Condition 4-4.

The collapse of the surface soil with holes could also be manifested as two phenomena, i.e., the sudden appearance of holes and the collapse of the surface soil and the first small-scale wet subsidence of the surface soil, and then the formation of holes and collapse. The former occurs under working Conditions 1-1 and 1-3, and the test phenomenon is shown in Figure 11; the latter occurs under the other remaining working conditions, and several test phenomena are shown in Figure 12. There was a sudden appearance of holes and collapse in the surface soil. Under Condition 1-1, as the water in the pipeline is continuously drained, the soil around the seepage hole is continuously eroded, causing oval ring cracks to appear in the upper surface soil. The soil collapsed instantaneously at 3 min and 30 s, revealing an oval hole. These are similar to the indoor test results obtained by Cheng et al. [26]. They show that when the buried water pipeline leaks, there will indeed be a cavity near the leakage hole. As the test progresses, the annular crack expands outward from the hole at the center, and the hole continues to expand. Owing to the water absorption and capillary action of soil, water flow will occur in the surface soil after collapse. When the water flow fills the hole, the soil around the hole collapses further, and ring-shaped cracks with larger scopes appear in the surface soil. The surface soil becomes wet in a small area first and then forms holes and collapses. Under Condition 1-2, cracks did not appear instantaneously, nor did the soil collapse to form holes. Instead, at 6 min and 30 s, annular cracks appeared and wet subsidence of the surface soil occurred, and no water flow was observed. As the test progresses, a leakage channel is formed inside the soil above the seepage hole, and the water flow continuously washes the soil along the leakage channel, causing many loose soil particles to accumulate under the surface. Eventually, the piping phenomenon occurs, the leakage channel expands and forms holes, and water flows in the surface soil. After the emergence of water flow, the soil around the holes collapsed further, as in the case where holes suddenly appeared and collapsed in the surface soil.

### 4.2. Monitoring Data Analysis

In data processing, due to the different test durations for each condition to reflect the soil deformation pattern during the test, an appropriate period was used for data analysis. The relative position of the cross section of each leakage point and the optical fiber measurement section in the length direction was measured and located. The measurement results are shown in Table 3.

#### 4.2.1. Surface Soil Deformation Analysis

**Figure 13:**
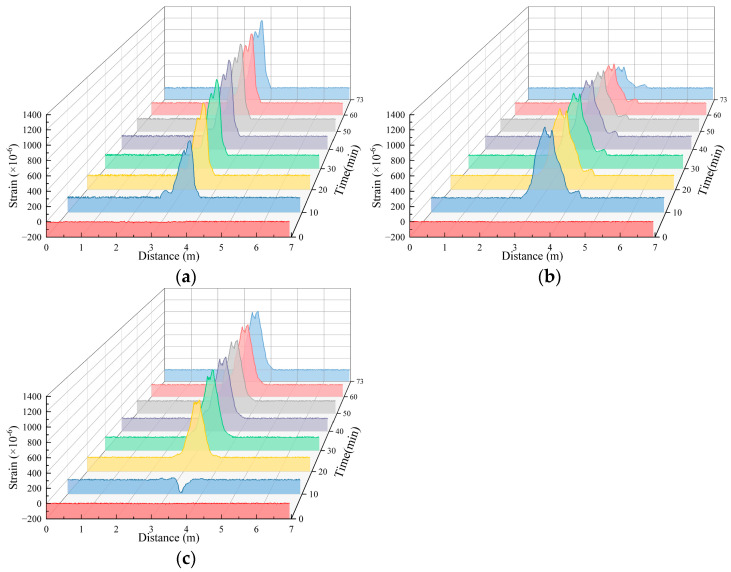
Working Condition 1-2. Fiber strain measurements for surface deformation monitoring. (**a**) Fiber strain measurements in Section A1; (**b**) fiber strain measurements in Section A2; (**c**) fiber strain measurements in Section A3.

Under different working conditions, the strain values obtained from the distributed fiber strain monitoring section of the soil surface after pipeline leakage exhibited several differences but essentially exhibited a similar distribution pattern. Because there are many test conditions, working Condition 1-2 was used as an example for analysis. The monitoring test results are shown in Figure 13; that is, the tensile strain was positive, and the compressive strain was negative.

Figure 13 shows that when the test of working Condition 1-2 was carried out, the water flow had a greater impact on the soil near the fibers in the A2 measuring section because the opening faced upward. The monitoring value of the fibers in the A2 measurement section was mainly tensile strain, which reached the maximum value at 10 min, and the tensile strain gradually decreased as the test progressed. The monitoring values of the fibers in the A1 measurement section also exhibited tensile strain, which reached a maximum at 50 min and then began to decrease. The monitoring values of the fibers in the A3 measurement section revealed compressive strain at 10 min and tensile strain for the remaining time. The tensile strain reached a maximum at 60 min of the test and then began to decrease. The reduction in tensile strain can be attributed to the following reasons: initially, during the onset of seepage, the soil mass begins to deform, and there exists a strong adhesive bond between the optical fiber and the soil, allowing them to deform conjointly. However, as soil particles are lost, the rearrangement of particles leads to a reduction in the adhesive bond between the optical fiber and the soil mass. This reduction in bond strength results in relative slippage between the optical fiber and the soil. Consequently, due to this relative slippage, the optical fiber is no longer able to fully capture the complete deformation of the soil mass, leading to a decrease in the strain values recorded by the fiber.

**Figure 14:**
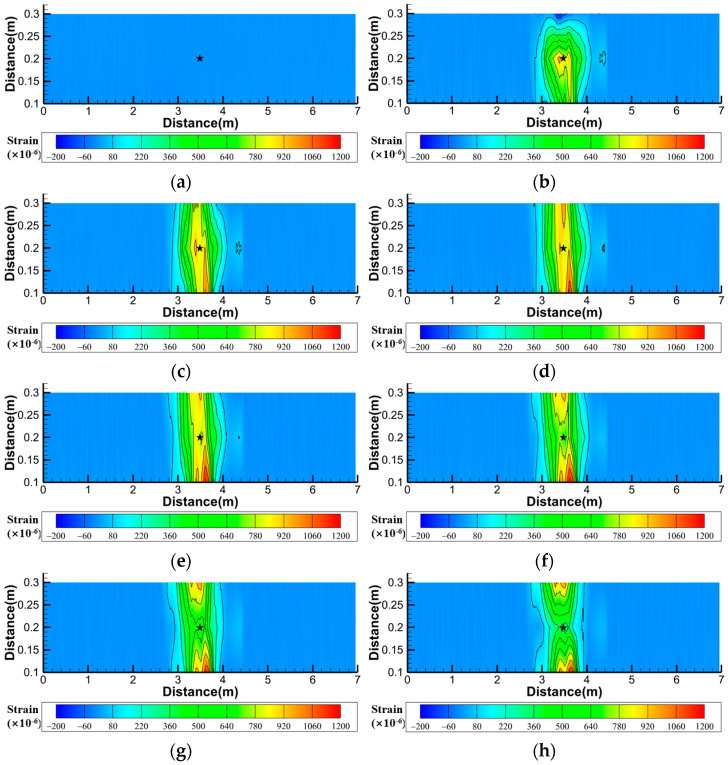
Contour map of strain of surface soil under different time in working Condition 1-2 (The asterisk indicates the center position of the pipeline seepage hole; x:y = 10:1). (**a**) Initial time; (**b**) 10 min; (**c**) 20 min; (**d**) 30 min; (**e**) 40 min; (**f**) 50 min; (**g**) 60 min; (**h**) 73 min.

To more intuitively demonstrate the soil strain variation process, Tecplot software (2023 R1) was also used to analyze the area enclosed by the A1, A2, and A3 fibers in Layer I of Figure 5, i.e., the rectangular area ABFE in Figure 5. The obtained contour plot of the soil strain field is shown in Figure 14.

Figure 14 shows that the surface deformation monitoring fiber can determine the direction of water flow seepage in the soil. Seepage first affects the fibers in the A2 measurement section and then the fibers in the A1 and A3 measurement sections. The measuring points with large changes in the three measuring sections of the fiber are not significantly different from the positions at the beginning of infiltration, whereas the tensile strains at the measuring points on both sides gradually increase, indicating that the seepage moving direction in each measuring section is the two directions of the parallel fiber. At 10 min, the strain value of the soil directly above the pipeline leakage location was the maximum, whereas the strain values on both sides were relatively small. After 20 min, the strain values of the soil on both sides began to increase, and the strain values of the soil directly above the pipeline leakage location began to decrease. After 50 min, the water flow on the surface soil had contacted the glass walls on both sides of the model box and began to spread to both sides, causing the strain value of the soil on both sides to start to decrease.

The monitoring results of the surface deformation monitoring fiber show that the fiber can locate pipeline leaks. In the soil deformation area, the strain fiber-measured values significantly changed. Most of the results obtained by fiber optics showed a “saddle” shape with two ends and a depression in the middle. These are similar to the results of Cheng et al. [26] and Wei et al. [25], who used water sac pumping to form a cavity to simulate the leakage of buried water pipelines. The monitoring results obtained by fiber optics display a “saddle” shape, with two peaks corresponding to the positions of the left and right shear sliding planes during ground subsidence [25]. Since this study simulated actual pipeline leakage processes rather than using water bag extraction methods, the similar fiber monitoring data indicate that after the leakage of underground water pipelines, the erosion of the water flow indeed forms a cavity inside the soil. A comparison of the monitoring results of the three monitoring sections revealed that when surface soil collapsed, if the water flow rate in the pipe was higher, the pipeline was buried deeper, the size of the seepage hole was larger, and the results of monitoring Sections A1 and A3 were better; if the seepage hole size was smaller, the monitoring results of Section A2 were better; and when the surface soil did not collapse, the monitoring result of the A2 survey section was better.

**Figure 15:**
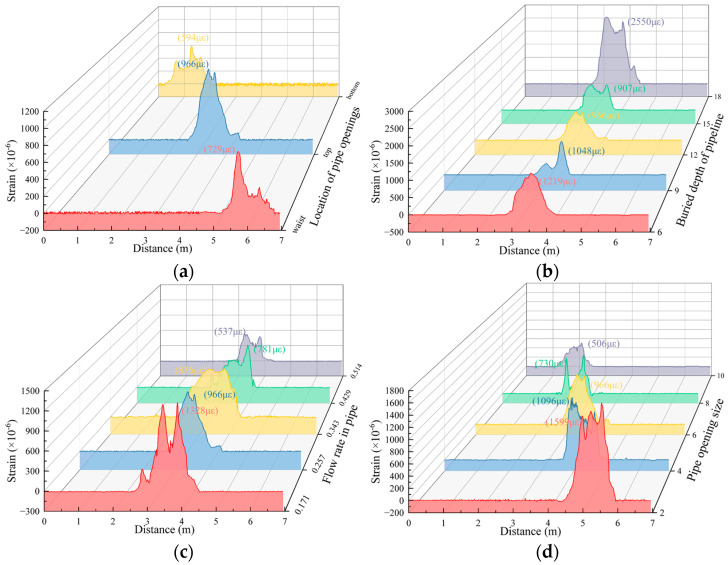
The fiber strain peak of A2 measuring section under different working conditions. (**a**) Different pipeline leakage locations; (**b**) different buried depth of pipeline; (**c**) flow velocity in different pipes; (**d**) different pipeline leakage size.

The experiment simulates the actual pipeline leakage process, so that the variation law of the strain peak of the optical fiber under different pipeline leakage conditions can be studied. Owing to the randomness of the locations of the leakage channels, not all the leakage channels in the tests were located directly above the leak holes in the pipeline but were slightly offset. Therefore, under the same working conditions, the peak generation times of the fibers in the A1 and A3 measurement sections were also the same. To analyze the variation pattern of the fiber peak strain under different variable tests, this paper analyzed the variation pattern of the peak value of the fiber in the A2 measurement section, as shown in Figure 15. The results are as follows: when the locations of pipeline leakage differ, the farther the location of the leakage opening from the surface, the smaller the peak strain. In addition, the “saddle” phenomenon was more obvious when the openings were located at the top and bottom of the tube. When the burial depth of pipelines is different, the “saddle” phenomenon becomes more evident with increasing burial depth. In a previous study [25,26], the deeper the water bladder was buried, the smaller the fiber strain monitoring value was after the formation of a cavity inside the soil. This finding is similar to the fiber monitoring pattern obtained by changing the burial depth of the pipeline in the present experiment. The monitoring result of working Condition 2-5 is too large, which is discussed in the following discussion. When the flow rate of water in the pipe is different, the higher the flow rate in the pipe, the smaller the peak strain. When the pipeline leakage size is different, the larger the pipeline leakage size, the smaller the peak strain. When the flow rate in the pipe is high or the leakage size is large, the leakage water scours the soil around the pipe with high kinetic energy, rapidly forming and expanding the leakage channel, which reduces the impact and erosion of the soil, resulting in a decrease in peak strain.

#### 4.2.2. Analysis of the Deformation of the Soil Under the Pipeline

**Figure 16:**
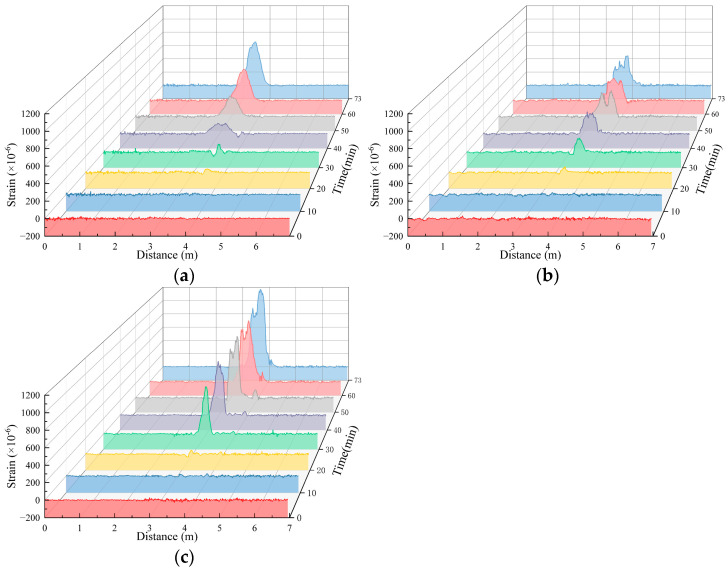
Working Conditions 1-2. The strain measurement uses fibers to monitor the deformation of the soil at the lower part of the pipeline. (**a**) Fiber strain measurement in Section B1; (**b**) fiber strain measurement in Section B2; (**c**) fiber strain measurement in Section B3.

Under different working conditions, the strain values obtained from the distributed fiber strain monitoring section of the soil deformation monitoring in the lower part of the pipeline after pipeline leakage exhibited several differences but essentially exhibited a similar distribution pattern. Here, working Condition 1-2 was also taken as an example to carry out the analysis. The monitoring results of the test are shown in Figure 16. The tensile strain was positive, and the compressive strain was negative.

Figure 16 shows that during the test of working Condition 1-2, significant tensile strain was monitored by the three measuring sections of the fibers, and the measured value of tensile strain increased during the test. The fiber monitoring value of the B1 measurement section changed significantly at 40 min, and the fiber monitoring values of the B2 and B3 measurement sections showed significant changes at 30 min. Because water flow will cause changes in the soil strain field after encountering the soil, the measurement can be based on different measurements. The monitoring value of the fiber segment significantly changes, which is used to determine the seepage direction of the water flow in the soil.

**Figure 17:**
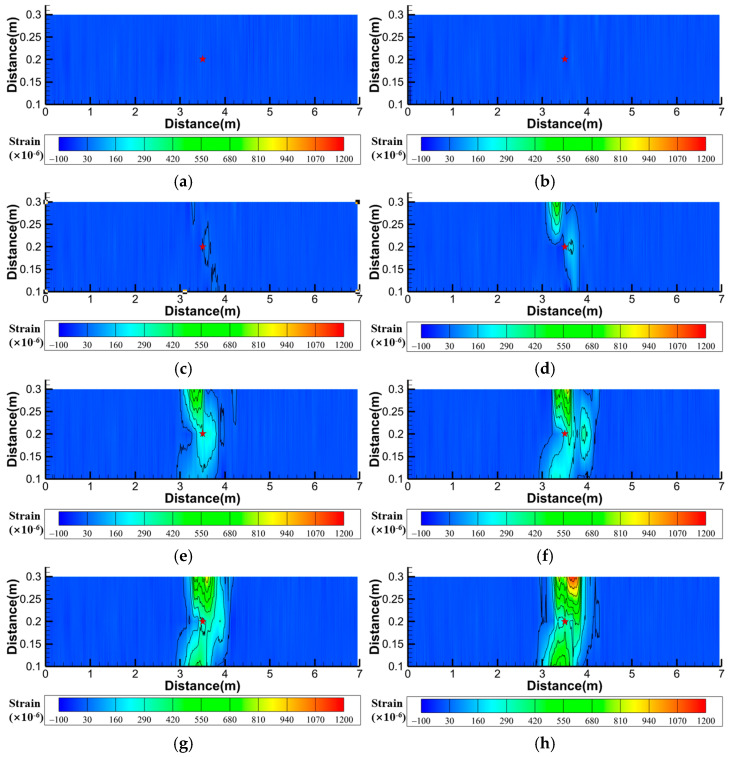
The strain contour map of the lower soil of the working Condition 1-2 pipeline at different times (The asterisk indicates the center position of the pipeline seepage hole; x:y = 10:1). (**a**) Initial time; (**b**) 10 min; (**c**) 20 min; (**d**) 30 min; (**e**) 40 min; (**f**) 50 min; (**g**) 60 min; (**h**) 73 min.

To more intuitively demonstrate the soil strain variation process, Tecplot software (2023 R1) was also used to analyze the area enclosed by the B1, B2, and B3 fibers in Layer II of Figure 5, i.e., the rectangular area A`B`F`E` in Figure 5. The obtained contour plot of the soil strain field is shown in Figure 17.

Figure 17 shows that seepage only gradually affected the soil around the soil deformation monitoring fiber in the lower part of the pipeline after 20 min; before 20 min, the strain value of the soil near the fiber essentially did not change. The soil deformation monitoring fiber in the lower part of the pipeline can also determine the direction of water flow in the soil. Figure 17d shows that at 30 min, the infiltration of water first affected the fibers in the B3 measuring section, and then, the tensile strain of the fibers in the B3 measuring section increased. The measuring points with large magnitude changes on the fiber at the B3 measuring section constantly deviated to point E, indicating that the movement direction of seepage in the B3 measuring section was the direction of EF; after 50 min, the infiltration of water flow affected the fiber at the B1 measuring section, as shown in Figure 17f. The tensile strain of the fibers in the B1 measurement section increases. The measuring point on the fiber in the B1 measuring section changed significantly from the position at the beginning of infiltration, whereas the tensile strain at the measuring points on both sides gradually increased, indicating that the seepage moving direction in the B3 measuring section comprised the AB and BA directions. The infiltration of water flow affected the fibers in the B2 measuring section for 40 min. At this time, under the influence of seepage in the B3 section, the seepage direction of the B2 measuring section was in the CD direction. However, as the seepage flow in the B1 measuring section gradually increased, the seepage in the B2 measuring section shifted slightly in the DC direction.

**Figure 18:**
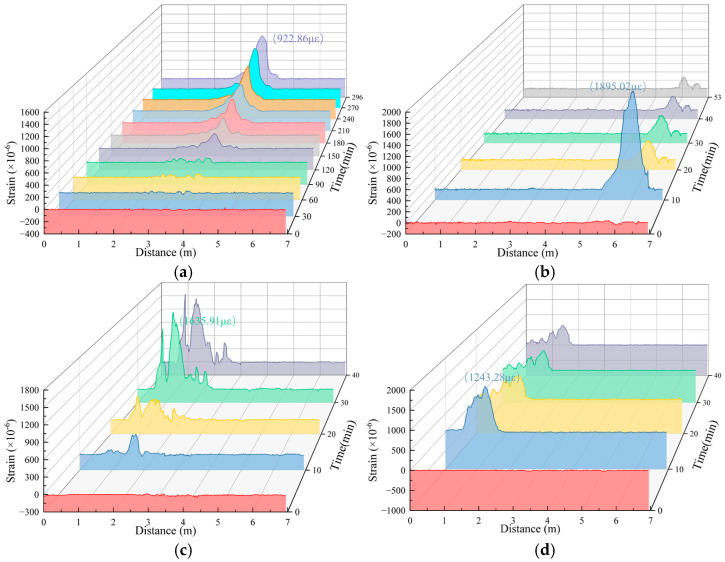
Optical fiber strain measurement of soil deformation monitoring under different working conditions. (**a**) Condition 1-1 B3 monitoring section monitoring optical fiber strain measurement value; (**b**) Condition 2-5 B1 monitoring section monitoring optical fiber strain measurement value; (**c**) Condition 4-5 B1 monitoring section monitoring optical fiber strain measurement value; (**d**) Condition 1-3 B3 monitoring section monitoring optical fiber strain measurement value.

The monitoring results using the soil deformation monitoring fiber at the lower part of the pipeline show that the strain fiber used to monitor the soil deformation at the lower part of the pipeline can also locate the location of pipeline leakage. In the soil deformation area, the measured value of the strain fiber significantly changes. A comprehensive comparison of the monitoring results of the three survey sections under each work condition reveals that the monitoring results of the B1 and B3 survey sections are better. The monitoring data of some working conditions are shown in Figure 18. A comparison of the monitoring data of the soil deformation monitoring optical fiber under the pipeline reveals that the results obtained by the optical fiber are not the same as those of the surface deformation monitoring optical fiber, showing a “saddle” shape with two towering ends and a middle depression and a variety of forms. More than half of the data roughly exhibited a unimodal distribution, and the data of some work conditions exhibited a multimodal distribution; there was a working condition that gradually increased the tensile strain or compressive strain, indicating that after the leakage of the underground water pipeline, the subsidence of the soil inside the soil was complicated by the erosion of the water flow.

#### 4.2.3. Analysis of Land Subsidence

**Figure 19:**
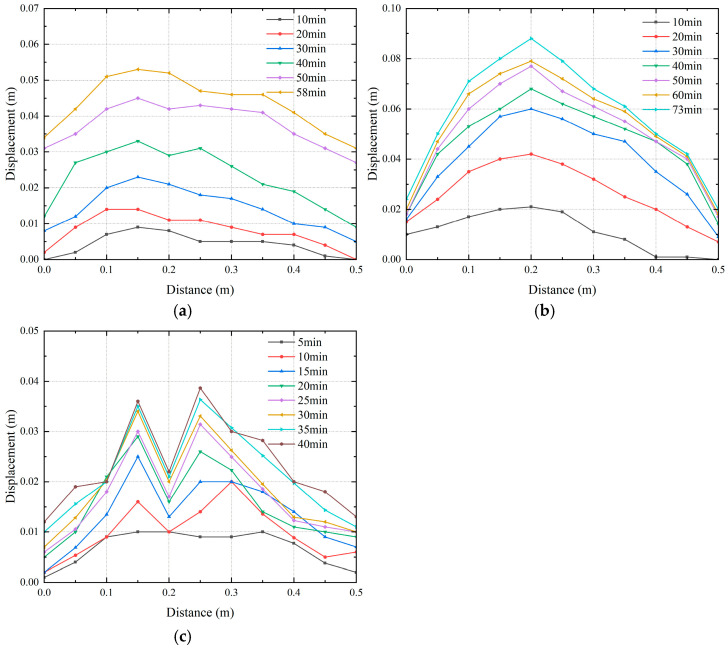
Settlement of surface soil when the seepage holes are located in different locations. (**a**) Ground settlement under working Condition 1-1; (**b**) ground settlement under working Condition 1-2; (**c**) ground settlement under work cases 1-3.

**Figure 20:**
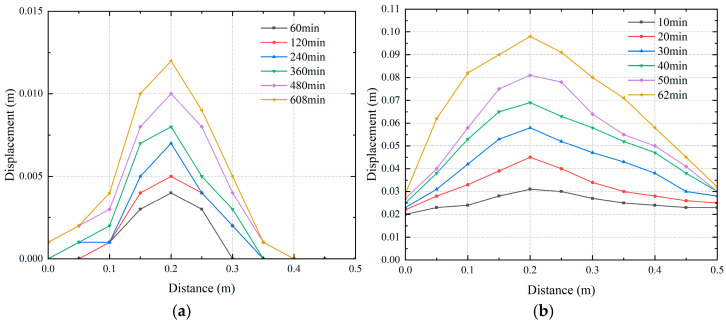

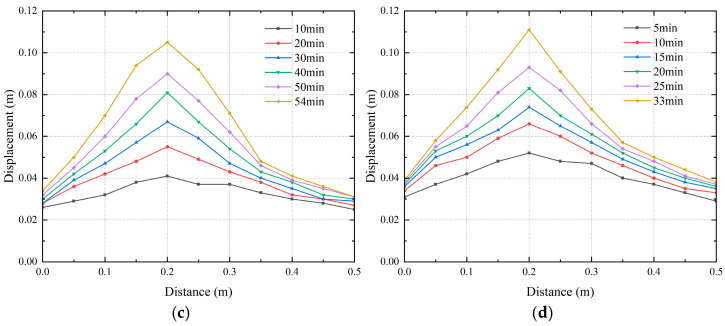
Settlement of surface soil under different pipeline flow rates. (**a**) Ground settlement under Work Case 3-1; (**b**) ground settlement under Work Case 3-3; (**c**) ground settlement under working Condition 3-4; (**d**) ground settlement under working Condition 3-5.

Leakage of buried water pipelines will cause settlement of the surface soil. When the pipe opens upward, the development of land subsidence is similar. In addition, considering that the maximum and minimum land subsidence values occur under different water flow rates in pipes, this paper focuses on the analysis of the ground surface under the conditions that the seepage hole is located in different locations and that the water flow rates in the pipes are different. The development of settlement is shown in Figure 19 and Figure 20. The scanning path of the laser rangefinder is shown as XY in Figure 5. The length is the width of the model box, and the position at 0.2 m is the axis of the pipe.

As shown in Figure 19, when the seepage hole is located at the pipe waist, the maximum displacement of the surface soil occurs at 0.15 m and the maximum settlement is 5.3 cm. When the seepage hole is located at the pipe top, the maximum displacement of the surface soil occurs at 0.2 m and the maximum settlement is 8.9 cm. The settlement curve roughly fits a triangular shape. When the seepage hole is located at the bottom of the pipe, the maximum displacement of the surface soil occurs at 0.25 m and the maximum settlement is 4.1 cm. The settlement of the surface soil corresponds to the test phenomena. When the seepage hole is located at the pipe top, due to water erosion and the leakage of soil particles into the pipe, the part of the surface soil with the largest settlement is located above the openings. When the seepage hole is located at the bottom of the pipe, the existence of the pipe causes the settlement amount of the soil on the upper side of the pipe to be small.

As shown in Figure 20, when the flow rate inside the pipe was changed, the location of the maximum settlement of the surface soil remained the same, i.e., it was still 0.2 m, and the settlement values of the soil surface exhibited a triangular distribution, which was consistent with the experiments of Zheng [5] and Cheng [26], whose results were similar. With increasing flow rate in the tube, the maximum settlement of the surface soil was as follows: 1.2 cm, 8.9 cm, 9.8 cm, 10.5 cm, and 11.1 cm. When the flow rate in the pipeline is low, there are no holes in the surface soil; therefore, the settlement is small. As the flow velocity inside the pipe increases, the water flow makes it easier to wash away the soil above the pipe, forming a funnel-shaped defect area and further increasing the settlement.

## 5. Discussion

### 5.1. Types of Ground Subsidence

When ground subsidence is caused by the leakage of a buried pipeline, uneven settlement of the soil will cause the soil arch effect. There are two types of ground subsidence modes: gradual ground subsidence [3,32] and sudden ground subsidence [33,34,35]. In progressive ground subsidence, the surface is always sinking, and the soil inside the ground subsides. The arch continuously forms and decomposes; in the case of sudden ground collapse, the cause of the collapse is the instability of the internal cavity, so land subsidence occurs instantaneously. The difference between these two subsidence types lies in whether a time-stable soil arch can be formed inside the soil [36]. If a time-stable soil arch can be formed, then the collapse mode is sudden settlement.

In this work, the monitoring results of the surface deformation monitoring optical fibers revealed a “saddle” shape, which is the same as the monitoring results of the optical fiber in the previous experiment of deliberately forming the internal cavity in the soil by pumping water with the water bladder, indicating that the collapse types in the present study were sudden. However, since the test was terminated when the distance between the seepage range of the water flow and the bottom plate of the model box was 10 cm, some working conditions revealed that no collapse of the holes occurred.

### 5.2. Coupling Between Fibers and Soil

When distributed fibers are used to monitor soil deformation, the coupling between the fibers and the soil is very important. The smooth protective layer on the outer surface of the fiber is prone to slipping relative to the monitored soil [37,38], affecting the reliability of the monitoring results.

**Figure 21:**
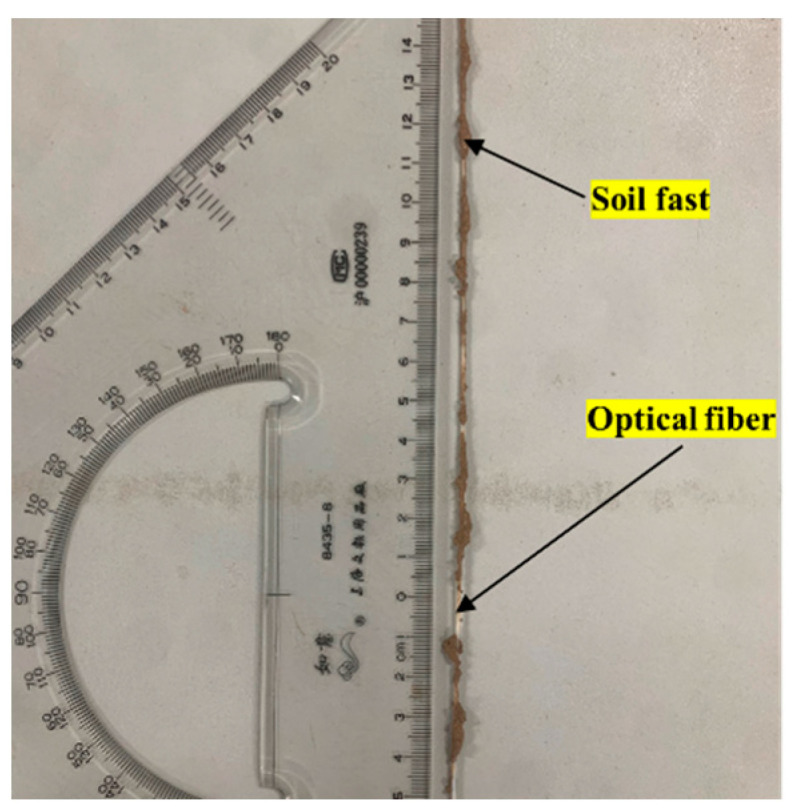
Phenomenon of fiber adhesion in soil.

**Figure 22:**
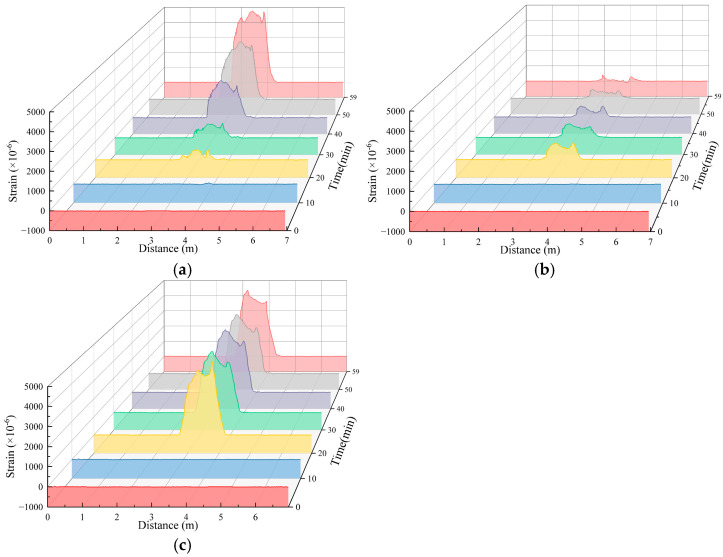
Working Condition 2-4. Fiber strain measurement for surface deformation monitoring. (**a**) Fiber strain measurement in Section A1; (**b**) fiber strain measurement in Section A2; (**c**) fiber strain measurement in Section A3.

In this work, the monitoring strain value for working Condition 2-5 was too large to be consistent with the actual pattern. During the disassembly of the fibers under working Condition 2-5, due to the change in the water content of the soil, part of the soil agglomerated into agglomerates and adhered to the fibers (Figure 21). In addition, the monitoring results of the surface deformation monitoring fiber under most working conditions in the test revealed that the monitoring results of the A1 and A3 sections were too large. When the fibers disassembled, the soil agglomerated into agglomerates and adhered to the fibers in the A1 and A3 sections, whereas the A2 section below the fibers in the measurement section was the pipeline seepage hole. Under the influence of water flow, the soil here is less likely to agglomerate into agglomerates and adhere to them. Taking working Condition 2-4 as an example (Figure 22), the monitoring results even exceed the monitoring results of fiber optic monitoring with the addition of anchor sheets reported by Cheng [26] and Wei [25], indicating that the coupling between the fiber and the soil is closely related to the behavior of the soil. The water content has a definite relationship, which warrants further study.

## 6. Conclusions

In this study, on the basis of the DFOSS technology of distributed optical frequency domain reflection, a model test of soil deformation monitoring caused by the leakage of buried water pipelines was carried out. Through the analysis of the measured strain data of optical fibers under different test conditions, the influences of factors such as leakage location, leakage size, pipeline burial depth, and water flow rate on the monitoring results of the soil strain field around the pipe were analyzed. The following conclusions were obtained:

(1) The measured strain values of the surface deformation monitoring fibers were mainly tensile strains, and the results were mostly “saddles” with towering ends and depressions in the middle. This pattern is consistent with other studies. We further explored the variation pattern of fiber peak strain under different leak sizes, flow velocities, burial depths, and leak locations; that is, the smaller the leak size is, the lower the flow velocity in the tube, and the lower the burial depth of the tube, and the peak is more obvious when the leakage location is at the pipe top.

(2) Most of the results obtained via optical fiber for soil deformation monitoring in the lower part of the pipeline exhibited a unimodal distribution, and the peak strain value was consistent with the leakage location.

(3) Compared with the monitoring results of different surveying sections, it is recommended that the fiber optic fibers be arranged above the pipeline to more effectively identify the location of ground subsidence.

(4) According to the sequence of time when significant changes in the measured strain values of the embedded optical fiber occur, the seepage direction can be effectively determined. This method provides a new technique for the early identification of leaks.

In summary, DFOSS technology effectively monitors the strain distribution of the soil during the collapse process, which is suitable for monitoring ground deformation caused by the leakage of buried water pipelines and can locate the leakage position of pipelines. The fiber monitoring effect was better and could accurately reflect the spatial distribution and variation pattern of the soil strain. DFOSS technology has broad application prospects in water diversion and water transfer projects. This technology can be used to monitor the health status of the soil around the water pipeline, prevent geological disasters such as ground subsidence, and provide important support for early warning of geological disasters.

## Figures and Tables

**Table 1:** Test equipment.

Equipment	OSI-C OFDR Demodulator	Sink	NZS-DSS-C07 Strain Measurement Fiber	Laser Range Finder	Flow Meter
Diagram	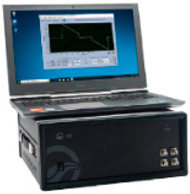	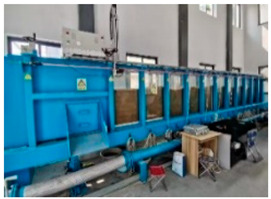	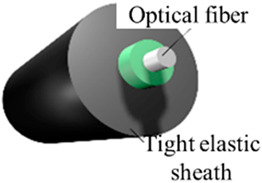	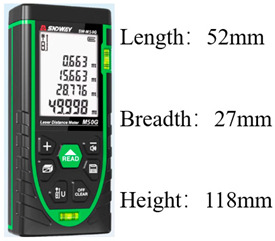	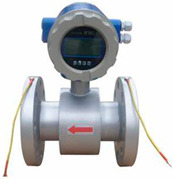

**Table 2:** Test conditions.

Work Condition Number	Hole Location	Pipeline Burial Depth (mm)	Pipe Flow Velocity (m/s)	Leakage Size (mm)
1-1	1	120	0.257	6
1-2	4	120	0.257	6
1-3	7	120	0.257	6
2-1	4	60	0.257	6
2-2	4	90	0.257	6
2-3	4	120	0.257	6
2-4	4	150	0.257	6
2-5	4	180	0.257	6
3-1	4	120	0.171	6
3-2	4	120	0.257	6
3-3	4	120	0.343	6
3-4	4	120	0.429	6
3-5	4	120	0.514	6
4-1	2	120	0.257	2
4-2	3	120	0.257	4
4-3	4	120	0.257	6
4-4	5	120	0.257	8
4-5	6	120	0.257	10

**Table 3:** Relative locations of the leakage points and the fibers.

Hole Location	A1 and B1	A2 and B2	A3 and B3
	Distance/m	Distance/m	Distance/m
1	8.53	11.53	24.73
2	7.68	12.38	23.88
3	6.83	13.23	23.03
4	5.98	14.08	22.18
5	5.13	14.93	21.33
6	4.28	15.78	20.48
7	3.43	16.63	19.63

## Data Availability

Data are contained within the article.
